# Non-Penetrance for Ocular Phenotype in Two Individuals Carrying Heterozygous Loss-of-Function *ZEB1* Alleles

**DOI:** 10.3390/genes12050677

**Published:** 2021-04-30

**Authors:** Lubica Dudakova, Viktor Stranecky, Lenka Piherova, Tomas Palecek, Nikolas Pontikos, Stanislav Kmoch, Pavlina Skalicka, Manuela Vaneckova, Alice E. Davidson, Petra Liskova

**Affiliations:** 1 Department of Paediatrics and Inherited Metabolic Disorders, First Faculty of Medicine, Charles University and General University Hospital in Prague, Ke Karlovu 2, 128 08 Prague, Czech Republic; dudakova.lubica@gmail.com (L.D.); vstra@lf1.cuni.cz (V.S.); lenka.piherova@lf1.cuni.cz (L.P.); skmoch@lf1.cuni.cz (S.K.); pavlina.skalicka@vfn.cz (P.S.); 2 Second Department of Medicine—Department of Cardiovascular Medicine, First Faculty of Medicine, Charles University and General University Hospital in Prague, U Nemocnice 2, 128 08 Prague, Czech Republic; Tomas.Palecek@lf1.cuni.cz; 3 UCL Institute of Ophthalmology, University College London, London EC1V 9EL, UK; n.pontikos@ucl.ac.uk (N.P.); alice.davidson@ucl.ac.uk (A.E.D.); 4 Department of Ophthalmology, First Faculty of Medicine, Charles University and General University Hospital in Prague, U Nemocnice 2, 128 08 Prague, Czech Republic; 5 Department of Radiology, First Faculty of Medicine, Charles University and General University Hospital in Prague, Katerinska 30, 128 08 Prague, Czech Republic; manuela.vaneckova@lf1.cuni.cz

**Keywords:** *ZEB1*, cornea, penetrance, loss-of-function

## Abstract

*ZEB1* loss-of-function (LoF) alleles are known to cause a rare autosomal dominant disorder—posterior polymorphous corneal dystrophy type 3 (PPCD3). To date, 50 pathogenic LoF variants have been identified as disease-causing and familial studies have indicated that the PPCD3 phenotype is penetrant in approximately 95% of carriers. In this study, we interrogated in-house exomes (*n* = 3616) and genomes (*n* = 88) for the presence of putative heterozygous LoF variants in *ZEB1*. Next, we performed detailed phenotyping in a father and his son who carried a novel LoF c.1279C>T; p.(Glu427*) variant in *ZEB1* (NM_030751.6) absent from the gnomAD v.2.1.1 dataset. Ocular examination of the two subjects did not show any abnormalities characteristic of PPCD3. GnomAD (*n* = 141,456 subjects) was also interrogated for LoF *ZEB1* variants, notably 8 distinct heterozygous changes presumed to lead to *ZEB1* haploinsufficiency, not reported to be associated with PPCD3, have been identified. The NM_030751.6 transcript has a pLI score ≥ 0.99, indicating extreme intolerance to haploinsufficiency. In conclusion, *ZEB1* LoF variants are present in a general population at an extremely low frequency. As PPCD3 can be asymptomatic, the true penetrance of *ZEB1* LoF variants remains currently unknown but is likely to be lower than estimated by the familial led approaches adopted to date.

## 1. Introduction

Posterior polymorphous corneal dystrophy (PPCD) is a rare autosomal dominant disorder manifesting as vesicular, band or geographic lesions and opacities of the innermost corneal layers; the endothelium and Descemet membrane [1,2].

The disease is caused by mutations in three genes, which all encode transcription factors that regulate epithelial-to-mesenchymal transition (EMT) and the converse process of mesenchymal-to-epithelial transition (MET), through a mutually inhibitory pathway [3,4,5]. The EMT/MET process has a vital role in normal development and tissue homeostasis, and its aberrant activation is related to cancer progression [6].

PPCD type 1 (PPCD1, OMIM # 122000) and PPCD type 4 (PPCD4, OMIM # 618031) are associated with pathogenic variants in the promoter of ovo-like 2 (*OVOL2*) and regulatory intronic region of grainyhead-like 2 gene (*GRHL2*) leading to ectopic expression of the encoded proteins [3,7]. *ZEB1* loss-of-function (LoF) alleles are known to cause PPCD type 3 (PPCD3, OMIM # 609141) [8]. To date 50 presumed LoF variants in *ZEB1* have been identified as disease-causing, including three full gene deletions and two large partial gene deletions. Most mutations are unique to the affected families; only two have been observed recurrently [9,10]. Notably, these reported variants are relatively evenly distributed across the gene, further supporting the hypothesis that PPCD3 results from *ZEB1* haploinsufficiency rather than any gain-of-function mechanisms that could be associated with a particular functional domain of the encoded protein [9,11].

PPCD3 has markedly variable phenotypic expressivity, even between members of the same family. Manifestation can range from congenital corneal edema to asymptomatic changes not affecting visual acuity [12]. In addition, three patients with *ZEB1* null alleles have previously been reported to have agenesis or hypoplasia of corpus callosum [13,14].

Available evidence suggests that approximately 5% of individuals harbouring pathogenic LoF variants in *ZEB1* do not display any detectable corneal abnormalities [2]. This indication of disease non-penetrance is, however, biased given that it has been derived by prior ascertainment of PPCD3 in patients and the examination of first-degree relatives. Thus, the true penetrance of ocular and brain pathology in subjects harbouring *ZEB1* LoF variants remains unknown as this would require population level screening or the ability to recall patients which most studies do not have the approved ethics for.

In this study, we interrogated in-house exome and genome sequencing data for *ZEB1* LoF variants in the Czech population and performed phenotyping in two individuals carrying presumably heterozygous LoF *ZEB1* variants.

## 2. Materials and Methods

In-house available massive parallel sequencing data, in total 3616 exomes and 88 genomes, obtained from patients investigated for suspected rare diseases affecting heart, kidney, nervous system, metabolism and eye in various research projects, as well as their unaffected relatives, were searched for the presence of LoF *ZEB1* alleles (Reference Sequence NM_030751.6). This search was performed because of our long-term research interest in PPCD and our ability to recall patients for further investigation due to our research consent agreement. DNA for these studies was extracted from venous blood using conventional protocols.

One individual, primary investigated for cardiomyopathy, was carrying a variant of interest. As he had been consented to be contacted for further research in case of possibly clinically relevant outcomes (approved by the Ethics Committee of the General Teaching Hospital in Prague, reference no. 55/18), he was invited for ocular examination. Validation of the variant and its segregation within the family was performed by conventional Sanger sequencing using DNA derived from buccal cells (Oragene OG-300, DNA Genotek, Ottawa, ON, Canada).

Ophthalmic examination included measurements of the best corrected visual acuity (BCVA) extrapolated to decimal values using Snellen charts and noncontact specular microscopy (Noncon ROBO Pachy SP-9000, Konan Medical Inc., Tokyo, Japan). The proband was also examined by standard magnetic resonance imaging (MRI) on a 3T MRI scanner. The protocol comprised 3D T1 magnetization-prepared rapid acquisition with gradient echo (MPRAGE), 3D fluid attenuated inversion recovery (FLAIR), 2D T2 weighted images (T2WI) and diffusion weighted images (DWI). Regional volumes of brain structures were measured to assess structural pathology.

GnomAD v2.1.1 sequencing data generated from 141,456 subjects (125,748 exomes and 15,708 genomes) of diverse origins not known to be affected by severe paediatric disease [15] was used to determine the general population frequency of *ZEB1* LoF variants. Only high confidence changes were taken into consideration. Variants flagged by LOFTEE (Loss-of-Function Transcript Effect Estimator) as low-confidence or with warning to use caution upon interpretation were not taken into consideration.

## 3. Results

The proband (II:1; Figure 1A), aged 47 years at ocular examination, was investigated by exome sequencing because of dilated cardiomyopathy manifesting at 42 years of age, without obvious cause including bioptically excluded myocardial inflammation. He was also noted to suffer, since the age of 36, from diabetes mellitus type 2 and arterial hypertension, since the age of 41. His cognitive functions appeared normal. The possible genetic cause of his heart failure has not been resolved.

**Figure 1:**
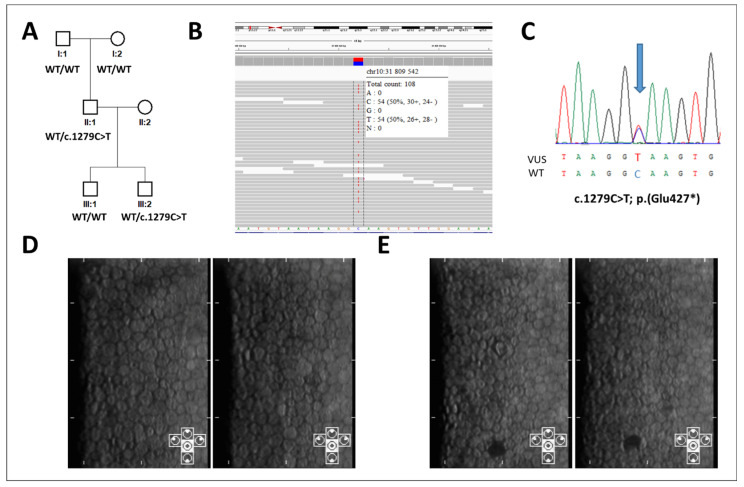
Genetic and clinical findings in two individuals with a predicted *ZEB1* loss-of-function variant. Pedigree of the family, individuals II:1 and III:2 carry the presumed loss-of-function (LoF) *ZEB1* variant: c.1279C>T; p.(Glu427*). (**A**) c.1279C>T as detected in the proband by exome sequencing from leucocyte-derived DNA (visualized in Integrative Genomics Viewer, aligned to Genome Reference Consortium Human Build 37); (**B**) and in his son as detected by Sanger sequencing from buccal cell derived DNA; (**C**) specular microscopy imaging of the corneal endothelium in the right and left eye of individual II:1; (**D**) and in the right and left eye of individual III:2; (**E**) the cells are of hexagonal shape with normal density for given age, there are no signs of posterior polymorphous corneal dystrophy. In individual III:2 one black dot in each eye is present, likely representing an isolated gutta.

Exome sequencing, however, revealed that he is a heterozygous carrier of a unique LoF variant c.1279C>T in *ZEB1* (NM_030751.6) predicted to cause an insertion of a premature termination codon; p.(Glu427*) (Figure 1B) presumed to be *de novo* as none of the parents were shown to carry the change. Familial segregation showed that his 7-year-old son, who was not known to have any general health or developmental abnormalities, also carries the variant in the heterozygous state (Figure 1A,C).

**Figure 2:**
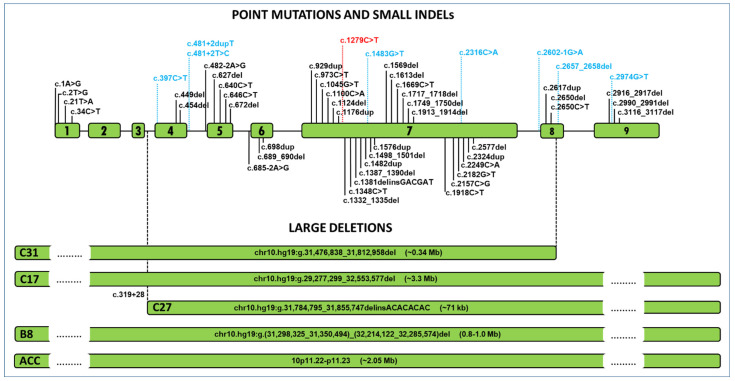
Predicted Loss-of-Function (LoF) variants observed in *ZEB1*. Posterior polymorphous corneal dystrophy type 3-associated mutations (*n* = 50) are shown in black. Variants identified in gnomADv.2.1.1 dataset predicted to result in *ZEB1* LoF are shown in blue (changes annotated as low confidence/dubious quality have been excluded). The presumed LoF variant detected in the Czech dataset comprising 7408 alleles is shown in red. All variants are predicted to either change transcript levels owing to large deletions, premature introduction of a stop codon, frameshift with premature termination of translation or alter splicing. Updated from Dudakova et al., 2019 [9].

Neither the proband nor his son reported, except for mild refractive errors, any eyesight-related symptoms and there was no family history of hereditary ocular disease. Ophthalmic examination did not detect any corneal or other ocular abnormalities characteristic of PPCD3. Both subjects had normal visual acuity with appropriate refractive correction (Table 1). Posterior corneal layers appeared by slit-lamp examination as normal with no opacities or lesions. The endothelial cells’ morphology and count were also within the normal range for given age group (Table 1, Figure 2) [16]. In the son, specular microscopy revealed one dark lesion in each eye, most likely representing isolated gutta. This finding is, however, nonspecific, not characteristic of the PPCD3 phenotype [12,17,18].

Brain MRI performed in individual II:1 showed a non-specific small hypersignal lesion in FLAIR and T2WI in the white matter of the right parietal lobe. The corpus callosum had normal shape, without pathological changes and its volume was in the 95% interval of healthy controls.

Excluding changes annotated as low confidence/dubious quality, gnomAD v2.1.1 lists five heterozygous carriers, each harbouring a unique presumed LoF variant located in exons with identified PPCD3-associated mutations. In addition, three further individuals have heterozygous variants within canonical splice sites that are likely to affect pre-mRNA splicing of *ZEB1* (Figure 2 and Supplementary Figure S1B).

A loss-of-function intolerant (pLI) score estimates the probability that a given gene is intolerant to haploinsufficiency [19]. A pLI ≥ 0.9 is widely used in research and clinical interpretation of cases with Mendelian inheritance and genes with this score are considered to be extremely intolerant to LoF [19]. The pLI score of *ZEB1* transcript ENST00000320985.10 corresponding to NM_030751.6, which has been used as reference sequence in reports on PPCD3-associated mutations, is 0.994 indicating high intolerance to LoF variants (Supplementary Figure S1A). In the gnomAD variation dataset, an observed and expected variant score (o/e constraint metric) has been introduced, in addition to pLI, to determine the probability that a given gene is intolerant to LoF variants. The closer the o/e is to zero, the more likely the gene is LoF-constrained. The o/e metric also incorporates a 90% confidence interval and as hard threshold the upper bound of the o/e confidence interval has been suggested to be <0.35 [20]. The o/e score of *ZEB1* (ENST00000320985.10) is 0.144 (CI 0.08–0.284), also suggesting marked intolerance to LoF (Supplementary Figure S1B).

## 4. Discussion

In order to assess the phenotypic penetrance associated with *ZEB1* LoF alleles, following a search of our local research variant database, one individual out of 3704 was found to carry a presumed LoF *ZEB1* allele in a heterozygous state.

Next, we performed clinical ocular examination in the proband and his son, both carriers of the heterozygous LoF variant c.1279C>T in *ZEB1* predicted to lead to an insertion of a premature stop codon at amino acid residue 427. This finding was considered unrelated to the underlying condition that triggered molecular genetic investigation in the proband. We also searched publicly available dataset gnomAD v2.1.1 alleles in order to assess the prevalence of *ZEB1* LoF variants in the general population.

Importantly, PPCD3-causing mutations have been reported both up- and downstream of the c.1279C>T variant identified in this study. Notably, this includes several disease-associated variants within exon 7 itself. Hence, the biological impact of this LoF variant cannot be dismissed due to alternative splicing of the transcript and the c.1279C>T variant would be predicted to be pathogenic. Notably, the two carriers investigated in the current study did not display abnormalities characteristic of PPCD3, i.e., no opacities, bands or vesicular lesions at the level of Descemet membrane and corneal endothelium. One isolated gutta detected in each eye by specular microscopy in the son was considered to be a non-specific finding often present in general population [17,18]. Although age-related penetrance cannot be fully excluded, the reported cases to date do not support this to occur in PPCD3 as the disease has been described in several young children [10,21]. Furthermore, non-penetrance has also been described in individuals of advanced age [8,12]. Collectively these findings suggest that age-related disease development is unlikely. As the variant was not found in the parents of the proband there is also a possibly of somatic mosaicism. However, given the equal and high coverage of wildtype and non-reference next generation sequencing reads spanning the variant of interest, mosaicism was not detected in the blood-derived gDNA (Figure 1B) [22].

As hypoplasia or agenesis of corpus callosum have previously been reported to be a rare phenotypic feature of PPCD3 [13,14] we also performed a brain MRI in the proband, which documented no structural pathology. His son did not undergo MRI; however, since he did not show any signs of developmental delay, cognitive impairment or had other neurological or head growth abnormalities, the chance of having unrecognized corpus callosum hypoplasia or agenesis is very low [23]. Interestingly, similarly to the current study, in one of the previously reported cases primarily investigated because of corpus callosum agenesis, the search for corneal disease was only initiated after obtaining results of molecular genetic investigation pointing at deletion of the entire *ZEB1* gene. Contrary to our study, typical corneal signs of PPCD3 were found [14].

Genes with pLI ≥ 0.9 comprise virtually all genes in which haploinsufficiency is associated with a severe human phenotype [19] and therefore null alleles are not expected to be present in control population. Interestingly, *ZEB1* (ENST00000320985.10) has a pLI score of 0.994 and o/e of 0.144 with upper confidence interval 0.284, suggesting that haploinsufficiency is not tolerated and, consistently, none of the point or small indel PPCD3-associated mutations reported so far are present in gnomAD v.2.1.1 Notably, this dataset includes eight heterozygous *ZEB1* variants predicted to induce LoF. However, given the lack of individual-level data available for the subjects recruited to this large project, they may in fact be affected by mild asymptomatic phenotypes such as PPCD3. Hence, in the absence of targeted phenotyping data it is not possible to know if any of LoF variants reported in gnomAD v.2.1.1 are PPCD3-associated.

Investigation of environmental and genetic modulators suppressing the effects of disease-causing mutations is important as it may lead to the identification of potential therapeutic targets. The apparent non-penetrance for LoF *ZEB1* variants observed not only in this study but also rarely in relatives from established PPCD3 pedigrees [8,12,24] may be attributed to *cis*-acting regulatory variants influencing the expression levels of wild-type alleles (e.g., inducing higher than average levels of expression from the wild-type allele compensating for the null allele). Another point that needs to be considered is the existence of alternate gene transcripts and understanding which are biologically relevant. Although *ZEB1* isoform 2 (NM_030751.6) has been used as the reference sequence in PPCD3 patients, to the best of our knowledge, direct evaluation of the various *ZEB1* transcripts in the corneal endothelium has not been performed. Finally, given that all genetic subtypes of PPCD reported to date are attributed to dysregulation of EMT/MET regulators (OVOL2, GRHL2 and ZEB1) [3,7,8], it is plausible to hypothesise that factors altering the expression of transcription factors in this pathway may also compensate for reduced *ZEB1* levels and thus explain incomplete penetrance in some individuals.

## 5. Conclusions

In summary, this study provides insight into the penetrance of LoF variants in *ZEB1*; causing a haploinsufficiency-related disease phenotype. We conclude that penetrance of *ZEB1* LoF alleles is likely lower than it has been estimated by the familial led approaches adopted to date. Our study also confirms that scores assessing LoF must be used carefully in the context of other available data [25].

## Figures and Tables

**Table 1:** Results of ocular examination in two individuals with heterozygous presumably loss-of-function variants in *ZEB1*.

Individual ID	Age (Years)/Gender	BCVA		Refractive ErrorDS/DC		ECD (Cells/mm^2^)	
		RE	LE	RE	LE	RE	LE
II:1	47/M	1.0	1.0	−3.25/−0.5 × 180°	−3.0/−1.25 × 135°	2538	2638
III:2	7/M	1.0	1.0	+0.25/−0.75 × 179°	−/−0.25 × 171°	3355	3125

BCVA = best corrected visual acuity, DC = dioptre cylinder, DS = dioptre sphere, ECD = endothelial cell density, LE = left eye, M = male, RE = right eye.

## Supplementary Materials

The following are available online at https://www.mdpi.com/article/10.3390/genes12050677/s1, Figure S1: Screenshots of gnomADv.2.1.1 page documenting *ZEB1* variants.
