# Supplementary material for: Non-Penetrance for Ocular Phenotype in Two Individuals Carrying Heterozygous Loss-of-Function ZEB1 Alleles
Source: Genes (Basel). 2021 Apr 30;12(5):677. doi: 10.3390/genes12050677 (PMC8146820; doi:10.3390/genes12050677)
Supplement: Supplementary file 1 [file genes-12-00677-s001.zip › genes-1061093-supplementary.pdf]

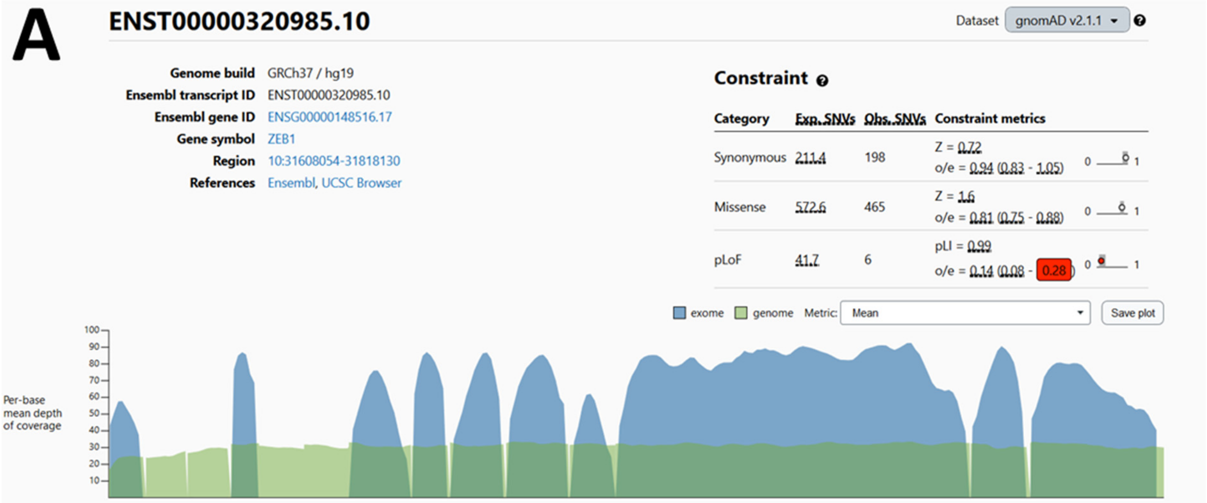

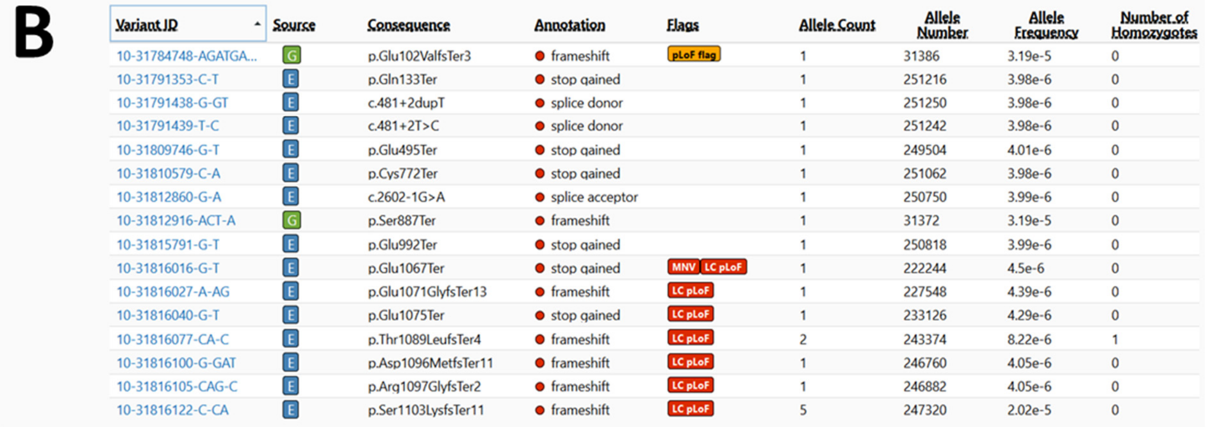

B

Variant ID	Source	Consequence	Annotation	Flags	Allele Count	Allele Number	Allele Frequency	Number of Homozygotes
10-31784748-AGATGA...	G	p.Glu102ValfsTer3	frameshift	pLoF flag	1	31386	3.19e-5	0
10-31791353-C-T	E	p.Gln133Ter	stop gained		1	251216	3.98e-6	0
10-31791438-G-GT	E	c.481+2dupT	splice donor		1	251250	3.98e-6	0
10-31791439-T-C	E	c.481+2T>C	splice donor		1	251242	3.98e-6	0
10-31809746-G-T	E	p.Glu495Ter	stop gained		1	249504	4.01e-6	0
10-31810579-C-A	E	p.Cys772Ter	stop gained		1	251062	3.98e-6	0
10-31812860-G-A	E	c.2602-1G>A	splice acceptor		1	250750	3.99e-6	0
10-31812916-ACT-A	G	p.Ser887Ter	frameshift		1	31372	3.19e-5	0
10-31815791-G-T	E	p.Glu992Ter	stop gained		1	250818	3.99e-6	0
10-31816016-G-T	E	p.Glu1067Ter	stop gained	MNV LC pLoF	1	222244	4.5e-6	0
10-31816027-A-AG	E	p.Glu1071GlyfsTer13	frameshift	LC pLoF	1	227548	4.39e-6	0
10-31816040-G-T	E	p.Glu1075Ter	stop gained	LC pLoF	1	233126	4.29e-6	0
10-31816077-CA-C	E	p.Thr1089LeufsTer4	frameshift	LC pLoF	2	243374	8.22e-6	1
10-31816100-G-GAT	E	p.Asp1096MetfsTer11	frameshift	LC pLoF	1	246760	4.05e-6	0
10-31816105-CAG-C	E	p.Arg1097GlyfsTer2	frameshift	LC pLoF	1	246882	4.05e-6	0
10-31816122-C-CA	E	p.Ser1103LysfsTer11	frameshift	LC pLoF	5	247320	2.02e-5	0

Figure S1. Screenshots of gnomADv.2.1.1 page documenting *ZEB1* variants. (**A**) pLI and o/e scores assessing intolerance to haploinsufficiency (**B**) Loss-of-function variants. Information shown is for the ENST00000320985.10 transcript that corresponds to NM_030751.6 used as the reference sequence in studies reporting on variants causing PPCD3. Source: https://gnomad.broadinstitute.org/.
